# Challenges in diagnosis and management of neutropenia upon exposure to immune-checkpoint inhibitors: meta-analysis of a rare immune-related adverse side effect

**DOI:** 10.1186/s12885-020-06763-y

**Published:** 2020-04-14

**Authors:** J. Boegeholz, C. S. Brueggen, C. Pauli, F. Dimitriou, E. Haralambieva, R. Dummer, M. G. Manz, C. C. Widmer

**Affiliations:** 1 Department of Medical Oncology and Hematology, University and University Hospital Zurich, Zurich, Switzerland; 2 Department of Dermatology, University and University Hospital Zurich, Zurich, Switzerland; 3 Institute of Pathology and Molecular Pathology, University Hospital Zurich, Zurich, Switzerland

**Keywords:** Immune-checkpoints-inhibitor, Neutropenia, Metamizole, Hematological side effects, Immune-related adverse events

## Abstract

**Background:**

Cancer immunotherapy via immune-checkpoint inhibition (ICI) by antibodies against cytotoxic T-lymphocyte-associated protein 4 (CTLA-4) and cell death protein 1 (PD-1) have significantly improved the outcome of metastasized melanoma and of a rapidly increasing number of other cancer types. The anti-tumor effect is often accompanied by immune-related adverse events (irAE). Hematological irAE, specifically neutropenia, are rarely observed. However, neutropenia is associated with high morbidity and mortality due to infection complications. Thus, early detection and treatment is crucial.

**Methods:**

We present the clinical course of two patients with severe neutropenia after ICI therapy and demonstrate the difficulty of the diagnosis when a comedication of metamizole, a well-known analgesic drug used to treat cancer pain, is present. Further, we provide a comprehensive descriptive and statistical analysis of published data on diagnostics, treatment and infection complication in patients with at least grade 4 neutropenia by a systematic database search.

**Results:**

Finally, 34 patients were analyzed, including the two case reports from our cohort. The median onset of neutropenia was 10.5 weeks after first ICI administration (interquartile range: 6 weeks). In 76% (*N* = 26), a normalization of the neutrophil count was achieved after a median duration of neutropenia of 13 days. In a subsample of 22 patients with detailed data, the infection rate was 13%, proven by positive blood culture in 3 cases, but 68% (*N* = 15) presented with fever > 38 °C. Treatment regime differed relevantly, but mainly included G-CSF and intravenous corticosteroids. Death was reported in 14 patients (41%), 3 of whom (9%) were associated with hematological irAE but only two directly associated with neutropenia.

**Conclusion:**

With an increasing number of cancer patients eligible to ICI therapy, the incidence of severe hematological toxicities may rise substantially over the next years. Clinicians working in the field of cancer immune therapies should be aware of neutropenia as irAE to provide immediate treatment.

## Background

Cancer immunotherapy by immune-checkpoint inhibition (ICI) of the cytotoxic T-lymphocyte-associated Protein 4 (CTLA-4) and programmed cell death protein 1 (PD-1) and its ligand (PD-L1) has significantly improved the treatment of metastasized melanoma [1]. It is also successfully being used to treat patients with advanced stage lung cancer, lymphoma, kidney and bladder cancer and an increasing number of other cancer types [2]. The physiological role of CTLA-4 and PD-1 mediates protection against autoimmunity and promotes self-tolerance [3]. By blocking CTLA-4 or PD-1 function, the host immune response against cancer cells is enhanced. However, due to the activation of the immune system, a wide spectrum of immune-related adverse events (irAEs) may occur. The organs most affected are skin, lungs, intestine, endocrine glands and liver, but irAEs can also occur in several other organ systems [4, 5]. The combination of CTLA4 and PD1 blockade increase the anti-tumor efficacy but, concurrently, also the rate of irAEs [6]. While hematological side effects have been rarely reported, the consequences of bone marrow dysfunction resulting in severe neutropenia is potentially lethal due to the high risk of bacterial or fungal infection, especially if neutropenia is prolonged [7]. As the overall incidence of neutropenia as irAE in cancer patients treated with ICI-therapy is low [8], only a few articles with patient data are available in the literature [9–32]. Some of these cases have been summarized in a recent descriptive observational study on hematological irAE [33]. Generally, the treatment procedure of irAE consists of discontinuation of the ICI therapy and administration of corticosteroids [34]. This therapy regimen could be contraindicated for patients with severe neutropenia, as the application of corticosteroids in this context might further increase the risk of infection [35]. Neutropenia as irAE in the hematopoietic system is multifaceted and the exact pathophysiological mechanism leading to bone marrow failure is still unknown. While anticancer drugs like chemotherapy are well recognized as causes of neutropenia, there are other drugs used in cancer treatment, that may cause neutropenia. This can lead to difficulties in determining the causative drug of the neutropenia as well as its treatment. In general, severe neutropenia as an adverse drug reaction is well known, for example in the case of metamizole (Novaminsulfon, Dipyrone), a popular and well-known antipyretic and analgesic drug. The treatment of drug-induced neutropenia consists of G-CSF and, if signs of infection are present, empiric antibiotic treatment with reconstitution within a few days [36]. However, several countries, such as the United States and the United Kingdom, have withdrawn metamizole from the market due to this adverse side effect. In most parts of Europe and Latin America, it is still broadly administered and successfully used to treat cancer pain [37]. Therefore, the combination therapy of metamizole and ICI is not so far-fetched and neutropenia in these patients poses challenges in diagnosis and treatment. We present the clinical course of two patients who were given this particular drug combination, with the documented reproducible irAE of severe neutropenia due to the ICI therapy in one of the cases. We supplement this report with a retrospective clinical data analysis of published clinical courses of patients with neutropenia as irAE after ICI treatment.

## Methods

### Patients selection

Adult patients with severe grade 4 neutropenia probably or certainly associated to every kind of ICI were independently searched by two authors, using PubMed search (http://www.ncbi.nlm.nih.gov). All case reports published in English language before the 14th of November 2019 were included. The terms “neutropenia”, “leucopenia”, and “pancytopenia” were combined (“AND”) with one or more of the following words: “immune checkpoint inhibitor”, “cytotoxic T-lymphocyte-associated Protein 4”, “anti-programmed cell death 1”, “anti-programmed cell death ligand 1”, “ipilimumab”, “nivolumab”, “pembrolizumab”, “atezolizumab”, “avelumab”, and “durvalumab”. Data on age, cancer disease, dosage and type of ICI, onset and duration of neutropenia, salvage therapy, other irAE, infection complications, blood and bone marrow results were collected and analyzed as described by the authors. The two patients described in this manuscript provided their informed consent; procedures were in accordance with the ethical standards of the responsible committee on human experimentation and with the Declaration of Helsinki.

### Statistical analysis

Analysis were done using Mann-Whitney U or Kruskal-Wallis test and contribution of factors to fever was tested by chi-square test. Duration of neutropenia was analyzed with log-rank test; correlations were quantified by Spearman’s rho. Statistical analyses were performed using SPSS Version 20.0 and RStudio 1.2.

## Results

### Case presentation

**Fig. 1:**
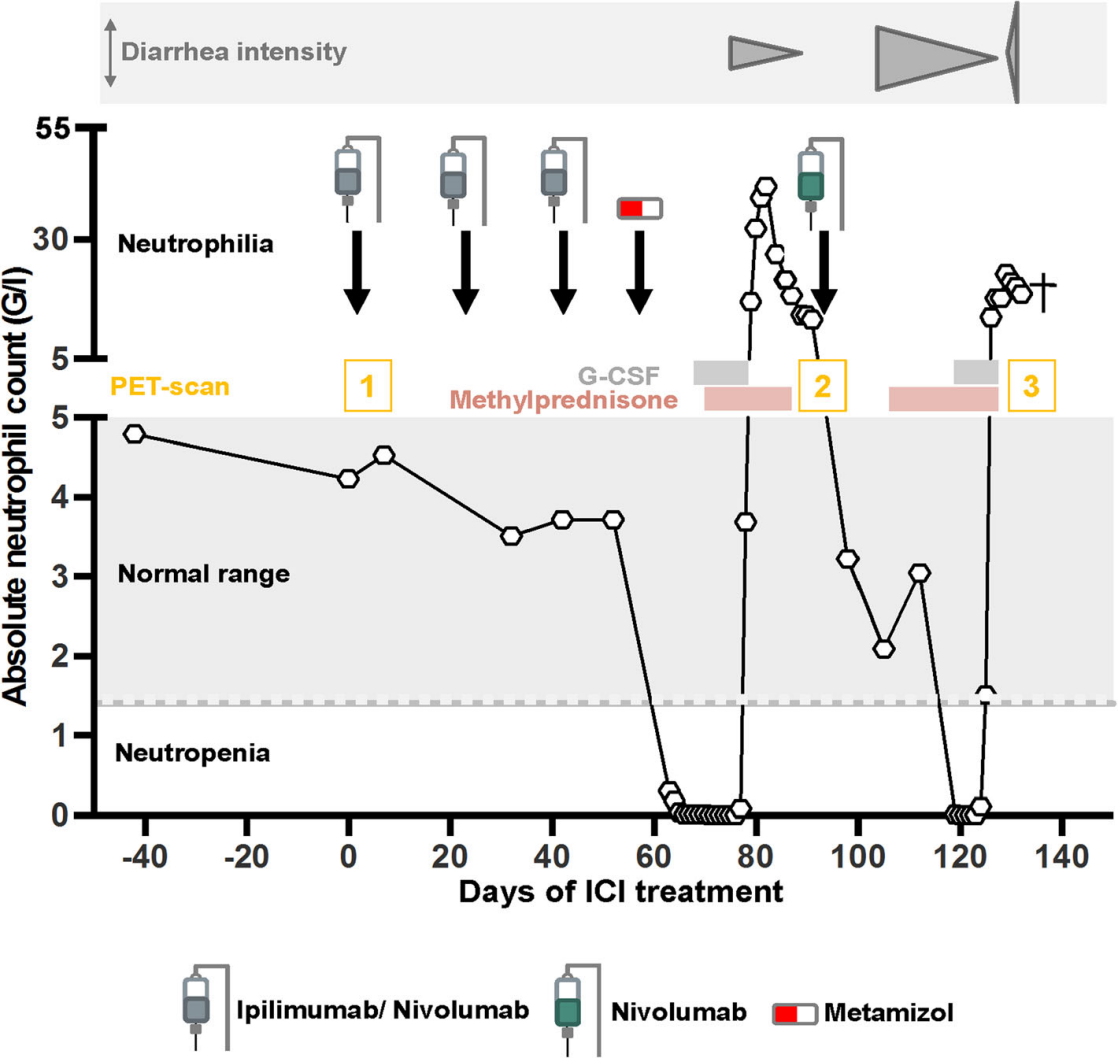
Timeline of patient 1. Neutrophil count over time following administration of ipilimumab, nivolumab and metamizol as well as subsequent interventions (application of G-CSF and methylprednisolone) are shown. The lower grey band marks the thresholds of neutrophils in the blood. The upper grey band shows concurrent diarrhoea that was intermittently active between and during events of neutropenia. Numbers in yellow indicate the time point of positron emission tomography (PET) with images shown in Fig. 2. The black cross marks the death of the patient

**Fig. 2:**
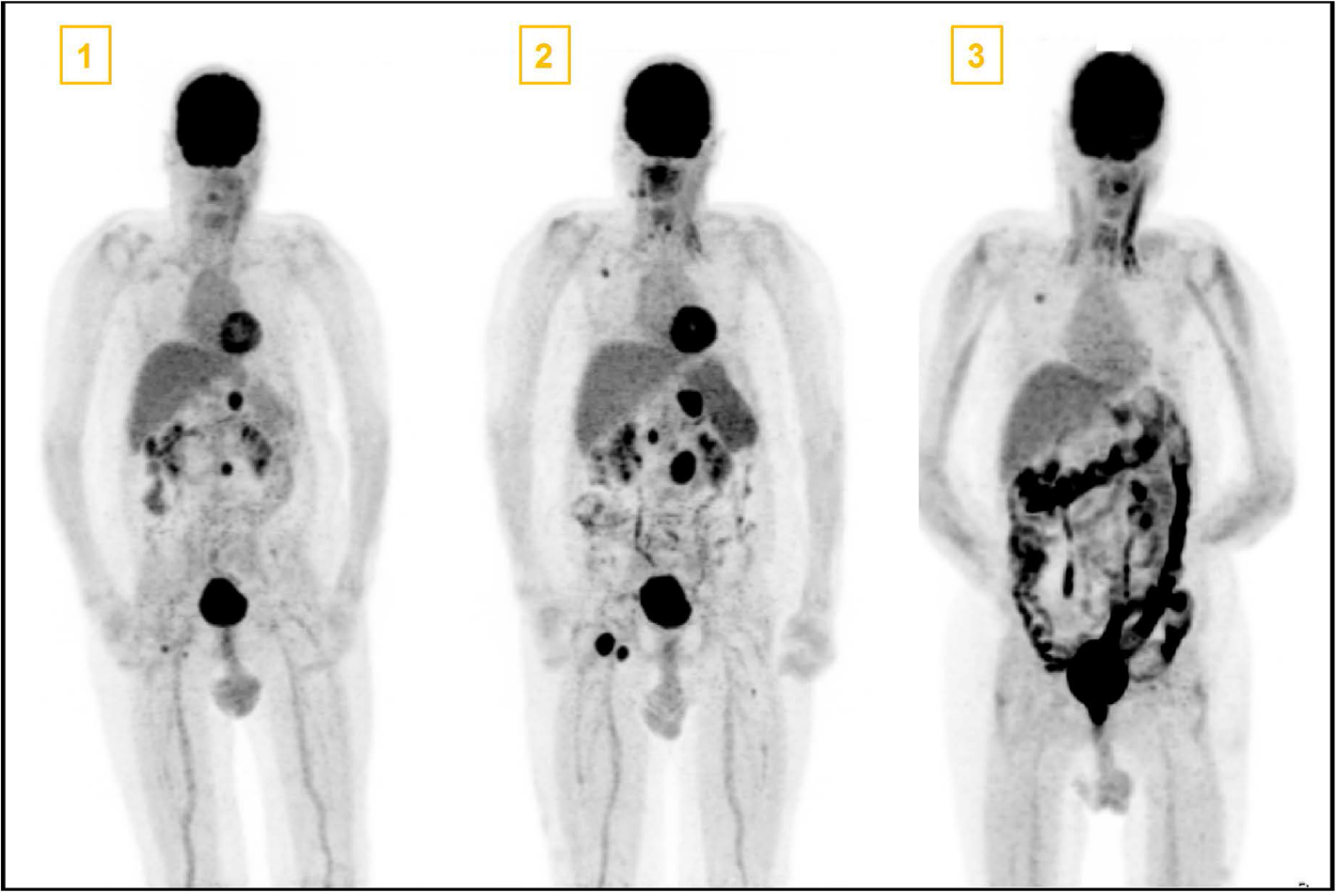
Positron emission tomography (PET) images at the time points as shown in Fig. 1. PET Number 1 depicts multiple metastasis (coeliacal, inguinal, pulmonary and retroperitonal). In PET image 2 a progression with coeliacal, retroperitoneal, paraaortal and inguinal lymph node but decreasing pulmonary melanoma manifestations was seen as mixed response after 3 cycles of ipilimumab and nivolumab therapy. PET image 3 shows complete remission of melanoma metastasis and a high activity in the whole colon due to massive Immune Checkpoint Inhibitor induced colitis. The patient received a port-a-cath system between the first and second PET scan

**Fig. 3:**
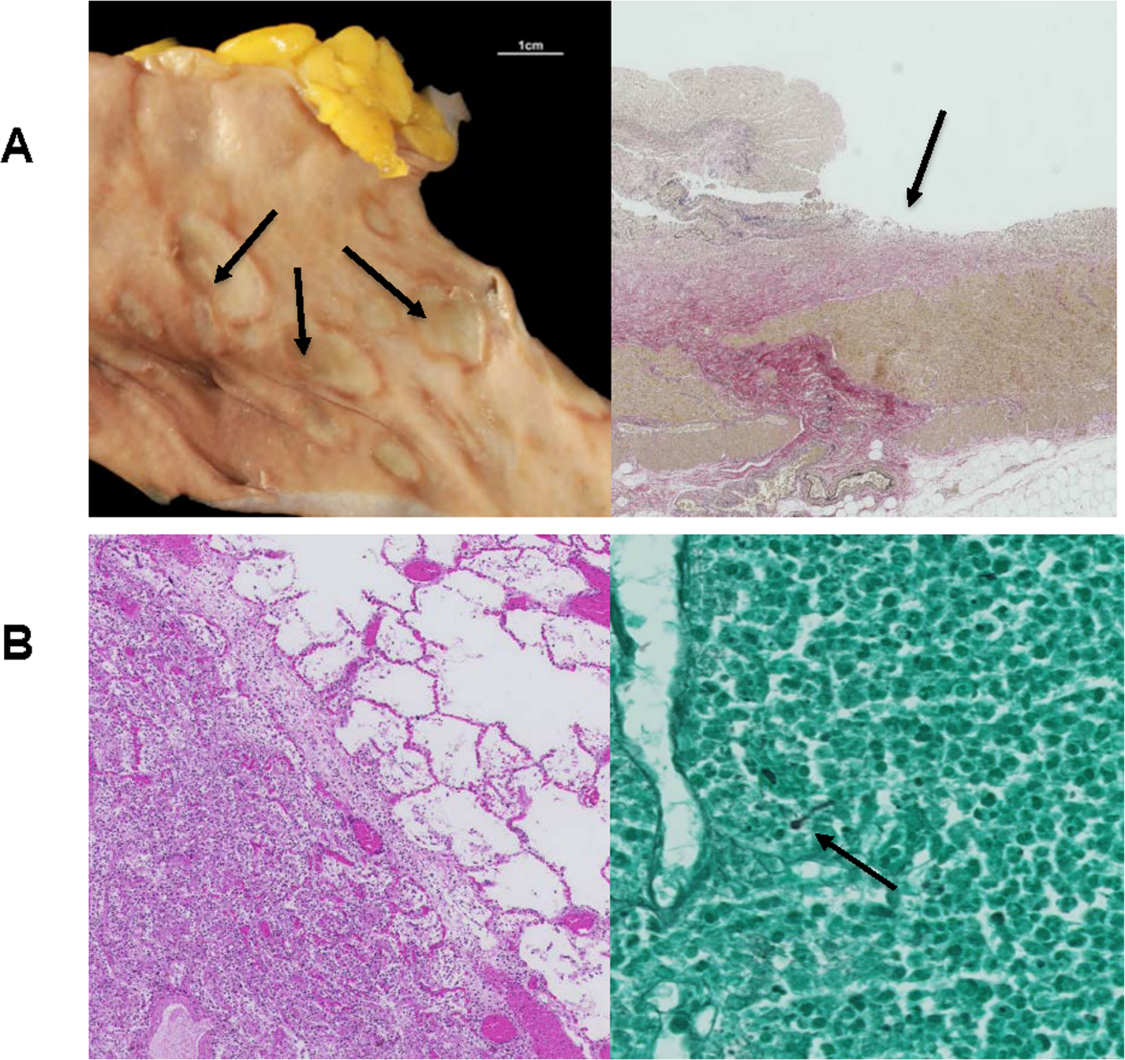
Autopsy results: Post mortem analysis revealed bowel wall injuries due to immune related colitis and fungal pneumonia. **a**: Indicated by arrow, macroscopic (left) and microscopic (right) skipped lesion in the intestine. **b**: Pulmonary fungal infiltration with microscopic demonstrated fungal hyphae (indicated by arrow))

Our first case, a 65-year old patient with *lentigo maligna melanoma* tumor stage IV (pT4b, N2b M1b), was initially treated with pembrolizumab (anti-PD1) and progressed in June 2016. A combination therapy with ipilimumab (anti-CTLA4) and nivolumab (anti-PD1) was initiated. At the beginning of the fourth cycle, a severe grade 4 neutropenia (0,01 G/l) with normal hemoglobin, thrombocyte and lymphocyte levels was documented. A detailed time line of medication application and side effects is shown in Fig. 1. A broad-spectrum antibiotic therapy was started and the patient was admitted to our ward. Due to the fact that the patient previously received a co-medication of metamizole (Novalgin®, Sanofi-Aventis AG) and clozapine (Leponex®, Novartis Pharma AG), with known side effects of severe neutropenia, a drug-induced cause of the isolated neutropenia was hypothesized. Bone marrow puncture at this time point revealed a nearly absent myelopoiesis without any other abnormalities. Histological analysis revealed small infiltrates of CD8 predominant lymphocytes. Stimulation with G-CSF (0,5 Mio. IE/kg daily) was immediately started at day 1. Due to persistent neutropenia for 9 days, corticosteroid treatment was added (methylprednisolone 1 mg/kg daily i.v.). Neutrophil recovery was reached 4 days later, overall 35 days after the last ICI application. Staging at this timepoint showed a partial response (Fig. 2). Upon interdisciplinary discussion with the patient and complete resolution of the neutropenia, he was re-exposed to nivolumab monotherapy with careful monitoring and without metamizole co-medication as metamizole was thought to be the cause of neutropenia. Four weeks later, a recurrence of grade 4 neutropenia (0.01G/l) occurred. Corticosteroid treatment and G-CSF stimulation as mentioned above were immediately restarted. The neutrophil recovered to normal values 1 week after supportive therapy was started. However, the patient suffered from a severe pulmonary infection and hemorrhagic diarrhea and died due to respiratory failure and septic shock 1 week later. The autopsy result showed an advanced fungal lung infection, most likely stemming from the repeated neutropenia, and a marked colitis with ulcerous skip lesion (Fig. 3). Histological findings from the colon showed intestinal stromal infiltration of lymphocytes, matching an immune-related cause. Strikingly, further findings confirmed a complete remission of the advanced melanoma.

The second case occurred in November 2018. A 56-year old patient with BRAFV600E metastatic melanoma stage IV (TxN3M1d), who was previously treated with chemotherapy (dacarbazine), BRAF inhibitor (vemurafenib), ipilimumab and pembrolizumab. After disease progression in July 2018, he was treated with nivolumab and additionally with anti-LAG-3 (NCT01968109). He presented with fever and an isolated grade 4 neutropenia (0.0 G/l) after 3 cycles of treatment and 26 days after the last infusion of nivolumab and anti-LAG-3. Additionally, he was treated with a maximal dose of metamizole (4x1g) due to cancer-related pain for 87 days. Immediate G-CSF application was started and 4 days later, prednisone 1 mg/kg/d was added, which was switched to methylprednisolone treatment another 4 days later. Intravenous Immunoglobulin (IVIG) (total dose of 1 g/kg) were applicated on days 9 to 11 after the diagnosis of neutropenia. Due to prolonged neutropenia, the initial broadband antibiotic therapy was supplemented with antimycotic therapy. Without any further complications, neutrophils recovered to normal values at day 15 after the initial episode. Bone marrow examination at day 4 confirmed an isolated complete absence of the myelopoiesis and showed a slight lymphocytosis of mostly CD8-positive T-cells. The following staging showed a progressive disease, ICI treatment was permanently discontinued and the patient received Dabrafenib and Trametinib (BRAF and MEK inhibitor) therapy, without any signs of intolerance. The patient was still alive in the last follow up, 3 months after ICI-treatment initiation. He was not re-exposed to metamizole.

### Systematic review

#### Literature search

**Fig. 4:**
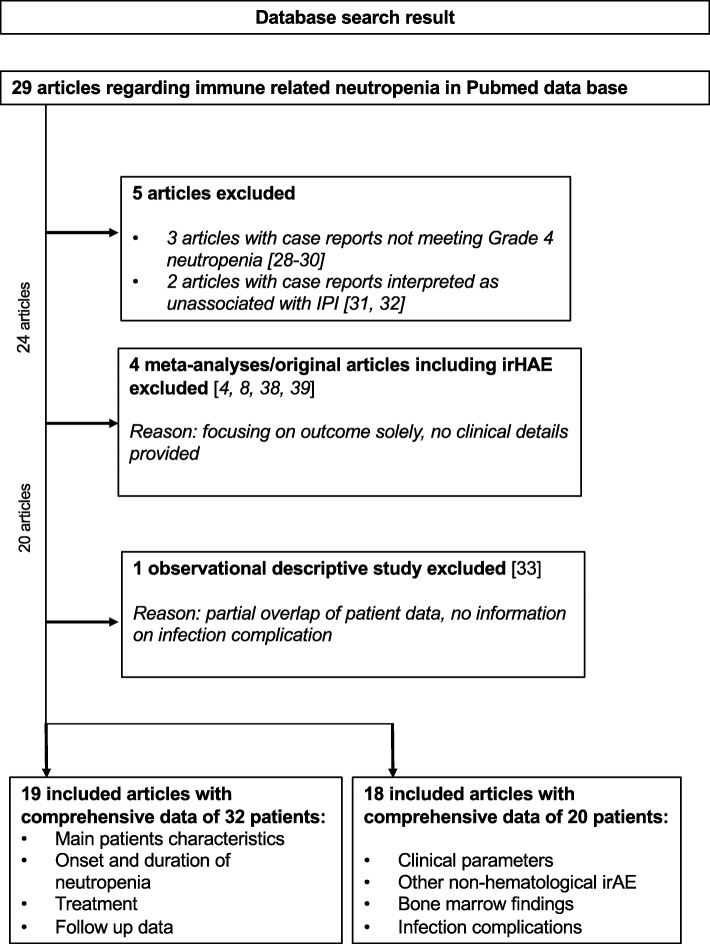
Patients selection flow chart meeting the search criteria. Final patient cohort. Overall 34 patients were selected. Due to a lack of detailed clinical information, an article with 9 patients could not be used for certain subanalysis

Twenty-nine articles matching our defined search criteria were identified. Four articles presenting meta-analyses with main focus of incidence of irAE were not included as detailed information were not provided [4, 8, 38, 39]. One observational study, which included previously published case reports, was excluded as it showed an overlap of case reports with our study but was further used for comparison of the results [33]. A second observational study presented summarized patient data but did not present reviewable information on detailed clinical course and it could only be partially included in the analysis [27]. Meeting the criteria of grade 4 neutropenia upon ICI exposure, 22 patients with detailed clinical course were identified through the PubMed search. Two case reports were excluded as we interpreted it to be unlikely that the reported neutropenia was caused by ICI [31, 32]. A detailed selection flow chart is shown in Fig. 4. Finally, we used 34 cases for the assessment of main characteristics and salvage therapy, duration of neutropenia and outcome and 22 cases for the analysis of non-hematological irAE on duration of neutropenia, clinical presentation and infection complication.

#### Meta-analysis: Main characteristics of patients with neutropenia upon ICI and additional irAE

Table 1 displays the main patient characteristics of the 32 published cases and the two cases described above. Isolated neutropenia was found for 11 patients (32%). In 5 cases the authors did not comment on other blood values, thus it can only be assumed that there was no other cytopenia present. Anemia was additionally reported in 16 cases (55%, median: 94 g/l). Of these, 7 patients presented an additional thrombocytopenia (all below 100G/l) and were therefore classified as an ICI-induced pancytopenia (20%). The combination of neutropenia and thrombocytopenia without anemia was not observed. Twenty cases reported on the presence or absence of additional irAE (rash, hepatitis, colitis; endocrine, pneumonitis) and for the others it was not documented. Isolated hematological irAE occurred in 9 cases (40%), while the others presented with prior or additional irAE.

**Table 1:** Baseline demographics, diseases, treatments, side effects, blood values and infection parameters of 20 patients in reviewed case reports (y = years, ICI = Immune Checkpoint Inhibitor, irAE = Immune-related side effects, n.a. = not applicable)

Patient	Age (y)	Cancer disease	ICI	irAE (besides neutropenia)	Neutrophil count (G/l)	Hemoglobin values (g/l)	Thrombocyte count (G/l)	Reported strain	Fever	Antibiotic therapy
1	42	Melanoma	Ipilimumab	Rush	0.3	108	202	None	Yes	Yes
2	74	Melanoma	Ipilimumab	Not documented	0	62	158	None	Yes	Yes
3	77	Melanoma	Ipilimumab	Endocrine	0	88	71	R. plantocola and E. cloacae	Yes	Yes
4	49	Melanoma	Ipilimumab	None	0	Normal	Normal	None	Yes	Yes
5	70	Melanoma	Ipilimumab	Rush	0.3	70	n.a.	None	No	n.a.
6	35	Melanoma	Ipilimumab	None	0	76	256	None	Yes	Yes
3	54	Melanoma	Ipilimumab	Rush, endocrine	0	n.a.	127	None	Yes	Yes
8	74	Lung cancer (adenocarcinoma)	Nivolumab	Rush, hepatitis	0	n.a.	n.a.	S. aureus	No	Yes
9	48	Melanoma	Nivolumab and ipilimumab	None	0	115	< 5	None	Yes	Yes
10	38	Primary mediastinal B-cell lymphoma	Nivolumab	None	0	n.a.	n.a.	None	n.a.	n.a.
11	73	Lung cancer (adenocarcinoma)	Nivolumab	Colitis	0	n.a.	n.a.	None	Yes	Yes
12	74	Lung cancer (adenocarcinoma)	Nivolumab	None	0	119	249	None	n.a.	n.a.
13	57	Glioblastom	Nivolumab	Rush	0	68	5	Moxarella catarrhalis	Yes	Yes
14	51	Melanoma	Nivolumab and ipilimumab	Endocrine	0	77	346	None	n.a.	n.a.
15	56	Lunge cancer (adenocarcoma)	Nivolumab	None	0.1	100	40	Fusarium solani	Yes	Yes
16	59	Melanoma	Nivolumab and ipilimumab	Rush, hepatitis, colitis	0.3	n.a.	n.a.	None	n.a.	n.a.
17	73	Lung cancer (adenocarcinoma)	Pembrolizumab	Not documented	0	n.a.	n.a.	None	Yes	Yes
18	82	Lung cancer (pleomorphic carcinoma)	Pembrolizumab	Pneumonitis	0.1	Normal	Normal	None	Yes	Yes
19	65	Melanoma	Nivolumab and ipilimumab	Colitis	0	103	227	None	Yes	Yes
20	56	Melanoma	Nivolumab and anti-LAG-3	None	0	144	288	None	Yes	Yes

#### Meta-analysis of onset and duration of neutropenia, clinical presentation and infection rate

**Fig. 5:**
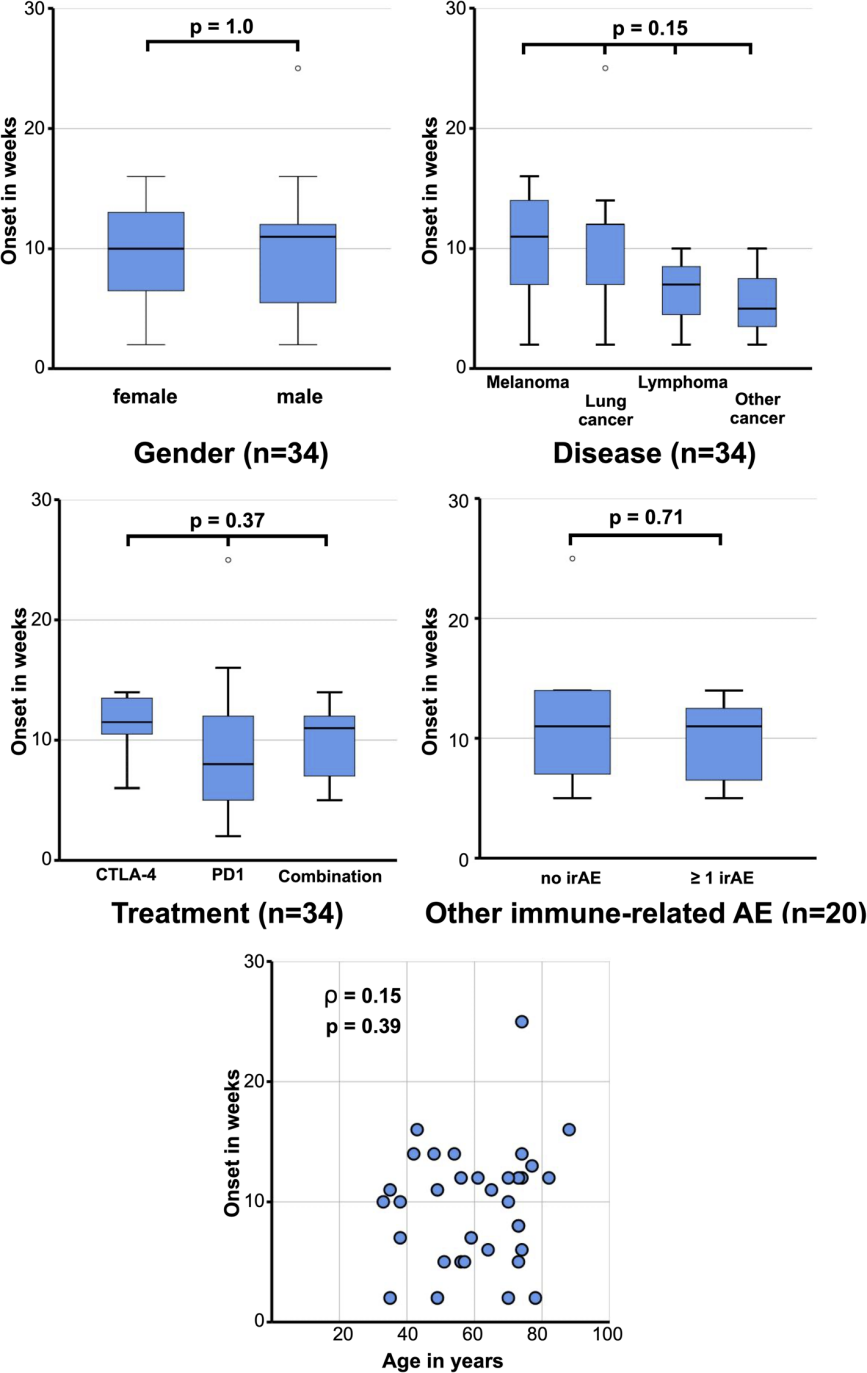
Boxplots analysis for onset of neutropenia in weeks and scatterplot for correlation with age. Gender (**a**), Diseases (**b**), Treatment (**c**), other immune-related adverse events (**d**) and age in years (**e**). A *p*-value of 0.05 was defined as significant

The median onset of the neutropenia was 10.5 weeks after the first dose of ICI therapy (min. 2.2, max. 25.4, interquartile range: 6 weeks). Adapted to the last ICI infusion the median time until neutropenia was documented after the last given dose was 12 days (min. 3, max 26, interquartile range 8 days). Neutropenia occurred mostly between the third and fourth cycle of the therapy (min. 1, max. 11 cycles, median 3.0 cycles). There was no statistical influence on time of onset of neutropenia regarding age or sex, underlying cancer disease, type of ICI treatment or additional presence of other non-hematological irAE (Fig. 5). Anemia was associated with a shorter onset of neutropenia (r = 0.49; *p* = 0.03), while thrombocyte values showed no statistically significant correlation (*p* = 0.22) (Additional Fig. 1). Further clinical details were available for 22 patients. The majority of them (*N* = 15, 68%) had a clinical presentation with fever > 38 °C at the time point of diagnosis of neutropenia and all of them were treated with broadband intravenous antibiotics. Chi-Square tests showed no relationship of the presence of fever with any of the following factors: gender (*p* = 0.59), disease (melanoma, lung cancer, lymphoma, or other cancers; *p* = 1.00), ICI (anti-CTLA4, anti-PD1 or a combination of both; *p* = 0.69), other irAE (no other irAE or more than 1 irAE; p = 1.00), or onset of neutropenia (< 10 weeks or > 10 weeks; *p* = 0.07). Only three patients (13%) had a bacterial infection, which was confirmed by positive blood cultures. Fungal infection was never observed at the initial neutropenic state. Of 16 cases (72%), bone marrow examination at diagnosis of neutropenia or during treatment with corticosteroid or G-CSF was available in detail. All bone marrow findings, except two, showed complete absence of the myeloid lineage. In the two cases without hypoplastic myelopoiesis, the bone marrow puncture was done 4, respectively 5 days before complete resolution of the neutropenia was achieved. In the vast majority, lymphocytosis was present but only few demonstrated a CD8+ T-cell lymphocyte infiltration by immunohistochemistry. No bone marrow biopsy showed a metastatic infiltration of cancer cells.

#### Variety of salvage therapy performed and outcome

The treatment regimens of neutropenia as irAE differed substantially among the 34 patients. Analyses of these results are summarized in Table 2. Most common was the treatment with G-CSF (*N* = 31, 91%), mostly in combination with corticosteroids (*N* = 24, 70%). IVIG were used in 35% of the cases (*N* = 12). If IVIG seemed to be effective, the available patient data showed a rapid response of 4 days (median). In 5 cases, additional immunosuppression with anti-thymocyte globulin (ATG) (*N* = 2) and/or cyclosporine was administered (*N* = 3). The duration of neutropenia, defined as neutrophil recovery >1G/l, was 13 days (min. 2, max 195, interquartile range 9.5 days). The duration of neutropenia differed not significantly between patients receiving IVIG or another salvage therapy while a shorter duration in two patients without steroids was observed (*p* = 0.04, Additional Fig. 2), the latter possibly by a recovery before an initiation of any therapy for neutropenia. The vast majority (*N* = 26, 76%) demonstrated a normalization of the neutrophil count and no significant difference in response between patients with IVIG alone, or in combination of another salvage therapy (regardless of IVIG) was observed (Additional Fig. 3). For 8 patients, a relapse of the neutropenia during tapering or after the stop of corticosteroids was documented. Five patients, including our first case, were re-exposed to ICI and 4 of them (80%) demonstrated a relapse of neutropenia. Our patient died after re-exposition, the other patients survived. Despite a remarkable antitumor activity and severe side effects in some cases, we were not able to draw a conclusion about the response to ICI therapy due to the lack of reliable information and small number of patients. Death during observation period was reported for 14 (41%), two of those as a consequence of ICI-induced neutropenia including our first case with fungal infection and one due to brain hemorrhage during severe thrombocytopenia [16]. In our study, we documented 9% of hematological irAE-associated deaths.

**Table 2:** Characteristics of onset, duration, treatment and outcome of Immune Checkpoint Inhibitor induced neutropenia in 20 patients (ICI = Immune Checkpoint Inhibitor, G-CSF = Granulocyte-Colony Stimulating Factors, IVIG = Intravenous Immunoglobulins, irAE = Immune-related side effects, n.a. = not applicable)

	ICI treatment		Onset	Treatment of neutropenia					Neutrophil recovery			Outcome		
Patient [ref]	ICI (mg/kg)	Number of cycles (cycle interval in weeks)	Documentation neutropenia (days after last ICI application)	Corticosteroids (dosage)	CSF (dosage)	IVIG (dosage)	ATG (dosage)	Other salvage therapy (dosage)	Neutrophil recovery achieved	Duration of neutropenia (days)	Recovery (days after salvage start)	Death during observation	Cause of death	irAE related death
1 [9]	Ipilimumab (10)	4 (3)	14	Prednisone p.o.; (1mg/kg/d) then dexamethasone i.v. (8mg/q6h)	G-CSF (480μg daily)	Yes (1g/kg/d)	No	Cyclosporin (2x100mg/d)	Yes	8	8	No	n.a.	n.a.
2 [10]	Ipilimumab (3)	3 (3)	21	Methylprednisolone i.v.(2x 1mg/kg/q12h )	G-CSF (n.a.)	Yes (n.a.)	Yes (4 x 15mg/kg/d)	Cyclosporin (2x2.5mg/kg/d)	Yes	20	4	No	n.a.	n.a.
3 [11]	Ipilimumab (3 then 10)	4 (3)	8	Prednisone p.o. (1mg/kg/d)	G-CSF (10μg/kg/d)	Yes (1g/kg/d)	No	Romiplostin (1μg/kg/week)	Yes	37	n.a.	No	n.a.	n.a.
4 [12]	Iplimumab (3)	3 (3)	12	Methylprednisolone i.v. (2x 2mg/kg/d)	GM-CSF (n.a.)	No	No	No	Yes	10	10	Yes	Brain hemo-rrhage	No
5 [12]	Ipilimumab (5)	5 (2)	n.a.	Prednisone p.o. (1mg/kg/d)	GM-CSF (n.a.)	No	No	No	Yes	7	7	No	n.a.	n.a.
6 [13]	Ipilimumab (3)	3 (3)	14	Methylprednisolone i.v. (120mg/d)	G-CSF (480μg daily)	No	No	No	Yes	16	8	Yes	Progression	No
7 [14]	Ipilimumab (10)	4 (3)	14	Prednisone p.o. (60mg/d)	G-CSF (5μg/kg/d)	No	Yes (4 x 15mg/kg/d)	Cyclosporin (2 x 125mg/d)	Yes	22	9	No	n.a.	n.a.
8 [15]	Nivolumab (3)	2 (2)	14	Prednisone p.o. then i.v. (1.5-3mg/kg/d)	G-CSF (n.a.)	Yes (n.a.)	No	No	Yes	16	14	No	n.a.	n.a.
9 [16]	Ipilimumab (3) and nivolumab (1 then 3)	4 (3 then 2)	3	Prednisone p.o. (1mg/kg/d)	G-CSF (n.a.)	No	No	No	No	n.a.	n.a.	Yes	Brain hemo-rrhage	Yes
10 [17]	Nivolumab (3)	3 (2)	7	No	G-CSF (n.a.)	Yes (n.a.)	No	No	Yes	7	3	No	n.a.	n.a.
11 [18]	Nivolumab (3)	5 (2)	12	Methylprednisolone i.v. (1mg/kg/d)	G-CSF (n.a.)	No	No	No	Yes	7	7	Yes	Malignant cardiac arrythmia	No
12 [18]	Nivolumab (3)	11 (2)	24	Prednisone p.o. (1mg/kg/d)	G-CSF (n.a.)	Yes (n.a.)	No	No	Yes	2	2	Yes	Progression	No
13 [19]	Nivolumab (3)	2 (2)	4	Dexamethasone p.o. (2mg/2xd)	G-CSF (n.a.)	No	No	Eltrombopag (50-100mg for 3 days)	Yes	64	22	Yes	Unknown	n.a.
14 [20]	Ipilimumab (3) and nivolumab (1)	2 (2)	7	Methylprednisolone i.v. (75mg/d)	No	No	No	No	Yes	17	8	No	n.a.	n.a.
15 [21]	Nivolumab (3)	2 (2)	4	Methylprednisolone i.v. (500mg/d)	G-CSF (5μg/kg/d)	Yes (n.a.)	No	No	Yes	48	25	Yes	Progression	No
16 [22]	Ipilimumab and nivolumab (not documented)	2 (Not documented)	5	Methylprednisolone i.v. (2mg/kg/d )	G-CSF (n.a.)	Yes (1g/kg/d)	No	No	Yes	16	16	No	n.a.	n.a.
17 [23]	Pembrolizumab (not documented)	4 (2)	3	Methylprednisolone i.v. (n.a.)	G-CSF (n.a.)	Yes (n.a.)	No	Cyclosporin (n.a.)	Yes	12	12	No	n.a.	n.a.
18 [24]	Pembrolizumab (not documented)	3 (3)	22	No	G-CSF (75μg daily)	No	No	No	Yes	7	2	No	n.a.	n.a.
19	Ipilimumab (3) and nivolumab (3)	3 (3)	12	Methylprednisolone i.v. (80mg/d)	G-CSF (0.5/kg/d )	No	No	No	Yes	13	4	Yes	Sepsis	Yes
20	Nivolumab and anti-LAG-3	3 (3)	26	Prednisone (1mg/kg/d), then methylprednisolone (1mg/kg/12h )	G-CSF (0.5/kg/d )	Yes (0.5g/kg/d)	No	No	Yes	15	7	No	n.a.	n.a.
21 [25]	Nivolumab (3)	1	16	No	No	No	No	No	Yes	n.a.	n.a.	Yes	Progression	No
22 [26]	Pembrolizumab (200mg absolut)	4 (3)	14	Prednisone p.o. (80mg/d)	G-CSF (0.5/kg/d )	No	No	No	Yes	4	4	Yes	Sepss	No
23 [27]	Nivolumab (not documented)	4 (not documented)	n.a.	No	No	Yes (n.a.)	No	No	No	>30	n.a.	Yes	n.a.	Yes
24 [27]	Nivolumab (not documented)	1 (not documented)	n.a.	Prednisolon 1mg/kg/d	G-CSF (n.a.)	Yes (n.a.)	No	No	No	>73	n.a.	Yes	Cardiac	No
25 [27]	Nivolumab (not documented)	6 (not documented)	n.a.	Prednisone 1mg/kg/d	G-CSF (n.a.)	No	No	No	No	>91	n.a.	No	n.a.	n.a.
26 [27]	Pembrolizumab (not documented)	3 (not documented)	n.a.	Prednisone 1mg/kg/d	G-CSF (n.a.)	No	No	No	Yes	12	n.a.	No	n.a.	n.a.
27 [27]	Nivolumab (not documented)	5 (not documented)	n.a.	No	G-CSF (n.a.)	No	No	No	Yes	>195	n.a.	No	n.a.	n.a.
28 [27]	Nivolumab (not documented)	1 (not documented)	n.a.	No	G-CSF (n.a.)	No	No	No	Yes	>111	n.a.	No	n.a.	n.a.
29 [27]	Pembrolizumab (not documented)	1 (not documented)	n.a.	Prednisone 1mg/kg/d	G-CSF (n.a.)	No	No	No	No	12	n.a.	No	n.a.	n.a.
30 [27]	Nivolumab (not documented)	6 (not documented)	n.a.	No	G-CSF (n.a.)	No	No	No	Yes	3	n.a.	No	n.a.	n.a.
31 [27]	Nivolumab (not documented)	8 (not documented)	n.a.	No	G-CSF (n.a.)	No	No	No	No	>36	n.a.	Yes	n.a.	Yes
32 [27]	Nivolumab (not documented)	5 (not documented)	n.a.	Prednisone 1mg/kg/d	G-CSF (n.a.)	No	No	No	Yes	14	n.a.	No	n.a.	n.a.
33 [27]	Nivolumab (not documented)	1 (not documented)	n.a.	No	G-CSF (n.a.)	No	No	No	No	11	n.a.	Yes	Progression	No
34 [27]	Pembrolizumab (not documented)	8 (not documented)	n.a.	Methylprednisolone i.v. (n.a.), then prednisone	G-CSF (n.a.)	No	No	No	No	28	n.a.	No	n.a.	n.a.

## Discussion

Treatment with immune blockade of CTLA4 and PD1 cell surface proteins on lymphocytes and tumor cells results in remarkable anti-tumor activity [40, 41] but can be accompanied by severe irAE. The number of irAE will increase in the following years, due to the growing application success of ICI to multiple types of cancers. In line with this, a higher incidence of irAE against hematopoietic cells is conceivable.

Delanoy and colleagues have recently described an average time to onset of hematological irAE of 10.1 weeks, including 12 cases with neutropenia. These published case reports were also included in our case series. Our analysis of these 12 and additionally 22 other cases resulted in a comparable average onset of neutropenia as a hematological irAE of 11 weeks. This is translating to an occurrence mainly between the third or fourth cycle of any kind of ICI treatment. The median duration of neutropenia was 13 days, but it varied substantially. As a consequence of the retrospective nature of the data, possibly under-reported co-medications, and the inconsistent supportive treatment approaches, the duration of ICI-induced neutropenia must be interpreted with caution. Overall, 76% of the cases achieved a normalization of the neutrophils, which is in line with the finding of the published results of Finkel et al. [33], who have done a comparable analysis of 20 overlapping patients with our cohort [9, 15, 17, 18, 21, 25, 27]. Focusing on irAEs, they are suggested to arise from general immunologic enhancement and are often manageable by discontinuation of the ICI therapy and/or treatment with immunosuppressive drugs [34]. Potentially fatal outcomes warrant appropriate treatment of ICI-induced neutropenia. However, as recommended for irAEs, additional immune-suppressive drugs for patients developing neutropenia pose a treatment conflict, as the infection rate may be increased. Considering this, fever as a sign of systemic inflammation was one of the cardinal symptoms which led to the diagnosis of neutropenia overall. As a consequence, all of these patients received broadband antibiotic from the first day of diagnosis. Bacterial blood stream infection rate was documented in only 13% of the cases and was already confirmed before corticosteroid treatment was applied. Death due to infection associated with ICI-induced neutropenia happened as an exception. However, based on the data analysis presented here, treatment with G-CSF in combination with corticosteroids seems not to worsen outcomes concerning infection complications and presents an acceptable initial clinical treatment approach. An initial antifungal therapy does not seem to be mandatory, as the duration of ICI induced neutropenia was not prolonged in most of the cases. However, fungal infection should be kept in mind. A positive effect of IVIG has been observed in some, but not all cases, and should be used in combination with G-CSF and corticosteroids. Immunosuppression with cyclosporine and ATG might be effective, especially in patients with dysregulated effector T-cells [42]. On the basis of current knowledge, the use of immunosuppressive drugs for ongoing irAEs is controversial and still being discussed; therefore, prospective studies are needed [43]. Due to the limited data, it is unclear whether the standard of care therapy applied to ICI or the rate of spontaneous recovery of the neutrophils resolved the neutropenia, and therefore, it must be stated as a hypothesis.

Concurrent treatment with medications that can cause neutropenia is a diagnostic challenge in patients treated with ICI. Neutropenia is a known adverse side effect of drugs like metamizole and clozapine*.* The presence of the enzyme myeloperoxidase in neutrophil granulocytes leads to the oxidation of drugs into reactive metabolites and may function as a hapten, triggering an immune reaction [44, 45]. These reactions occur restricted to certain individuals and therefore are called idiosyncratic drug reactions (IDR). Data for increased IDR in patients who received ICI are not available, but patients are possibly at a higher risk for IDR when their ability to immune tolerance is altered [46]. Drug-induced agranulocytosis seems to occur in 95% of the cases within the first 60 days of treatment [47]. Adapted to our second case, ICI and not metamizole-mediated neutropenia seems to be more probable, because of the timepoint of occurrence (between the third and fourth ICI therapy cycle) and because metamizole was taken continuously in maximal doses for a long period of time, namely 87 days. Bone marrow findings mentioned a growth-arrest of the myelopoiesis at the level of promyelocytes for metamizole, which implicates that the dividing pool and not the maturating pool of the myelopoiesis is susceptible to the metamizole immune-related toxicity. In our case series, bone marrow findings, except for one, showed an almost complete absence of the myelopoiesis. One case showed increased promyelocytes presence, but this particular finding occurred in a bone marrow taken 5 days before complete hematological reconstitution was reached. This might implicate a rather physiological finding during hematological recovery than a growth arrest of myelopoiesis. However, it demonstrates that bone marrow finding is time point-dependent and cannot provide a reliable distinction between ICI- or metamizole-induced neutropenia. Further, bone marrow lymphocytosis was present in some but not all cases and its function as a surrogate marker for immune-mediated neutropenia is difficult to prove. There is an ongoing discussion about the underlying mechanism of irAE [48]. Besides a T-cell mediated effect, an antibody driven process is suggested for neutropenia as irAE. Finkel and colleagues propose an interpretation of the underlying mechanism depending on the presence of antineutrophil antibodies and bone marrow finding [33]. This approach harbors difficulties. Measurement of antineutrophil antibody is not a standardized diagnostic approach to this very limited patient population and the absence of definable time points of bone marrow puncture in relation to the duration of neutropenia and treatment start will probably lead to misinterpretation of the bone marrow findings. The rarity of specific events in many different clinical settings is difficult to overcome and prospective comparing studies are needed with a focus on morphological, serological and histochemical findings in drug- or ICI-induced neutropenia to clarify if there is a potential difference. Bone marrow puncture, however, should be recommended in suspected ICI-mediated neutropenia anyway to exclude bone marrow infiltration of metastases of the primary treated tumor, as then treatment approaches differ substantially.

## Conclusions

In conclusion, the majorities of irAE upon ICI therapy occur within the first 4 months of treatment, matching the time point of appearance of neutropenia in the reviewed cases. Therefore, neutropenia as irAE, even if rare, is a side effect a clinician should be aware of and blood value measurement is recommended at the beginning of each cycle and of special interest in patients presenting with fever under ICI therapy. As a treatment option of neutropenia in these patients, it seems to be safe to administer intravenous corticosteroids additionally to the G-CSF treatment, even in inconclusive cases with comedication probably causing neutropenia. Broadband antibiotic therapy should be given in all cases with neutropenic fever and the fact that neutropenia relapsed during dose reduction of corticosteroids, points towards a management strategy with frequent blood value control and a slow tapering of the steroids in the follow-up of these patients. However, further data is needed to understand the exact mechanism and risk factors for bone marrow failure upon ICI therapy, possibly leading to a more sophisticated treatment approach.

## Supplementary information

**Additional file 1.** Scatterplots with correlation of onset of neutropenia in weeks for Hemoglobin (A) and Thrombocytes (B). Significance threshold was defined as < 0.05.

**Additional file 2.** Kaplan-Meier curves for duration of neutropenia in days in a patient subpopulation Diseases (A) ICI Treatment (B) other immune-related adverse events = irAE (C). Neutropenia therapy with steroids (D), IVIG (+ Steroids) (E) and other secondary salvage therapy (+/− IVIG) (F). Significance threshold was defined as < 0.05.

**Additional file 3 **Kaplan-Meier curves for response of salvage treatments in days for IVIG alone (A) other salvage therapy (+/−) IVIG (B). A *p*-value of < 0.05 was considered significant.

## Data Availability

All data generated or analyzed during this study are included in this published article.

## Abbreviations

ATG: anti-thymocyte globulin
CTLA-4: cytotoxic T-lymphocyte-associated protein 4
ICI: immune-checkpoint inhibition
IDR: idiosyncratic drug reactions
irAEs: immune-related adverse events
IVIG: Intravenous Immunoglobulin
PD-1: cell death protein 1
