# Supplementary figures and images for: Challenges in diagnosis and management of neutropenia upon exposure to immune-checkpoint inhibitors: meta-analysis of a rare immune-related adverse side effect
Source: BMC Cancer. 2020 Apr 14;20:300. doi: 10.1186/s12885-020-06763-y (PMC7155336; doi:10.1186/s12885-020-06763-y)

Additional file 1

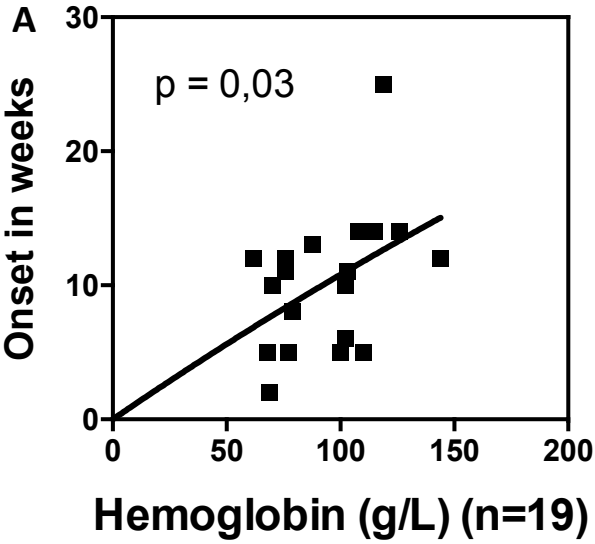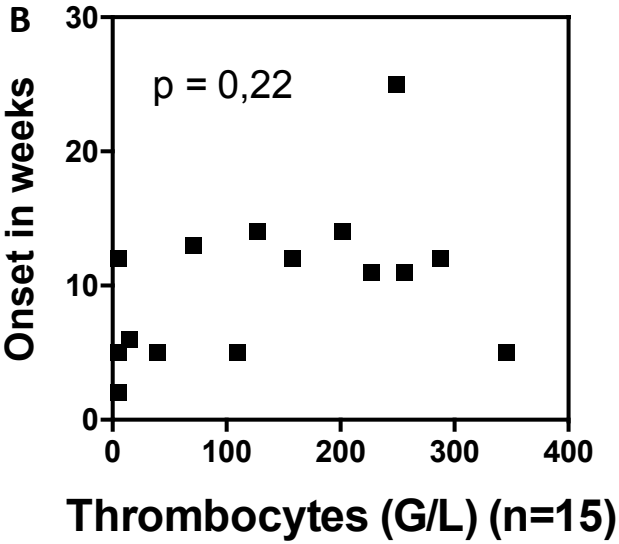

Supplement: Supplementary file 1 — Additional file 1. Scatterplots with correlation of onset of neutropenia in weeks for Hemoglobin (A) and Thrombocytes (B). Significance threshold was defined as < 0.05. [file 12885_2020_6763_MOESM1_ESM.pdf]

## Additional file 2

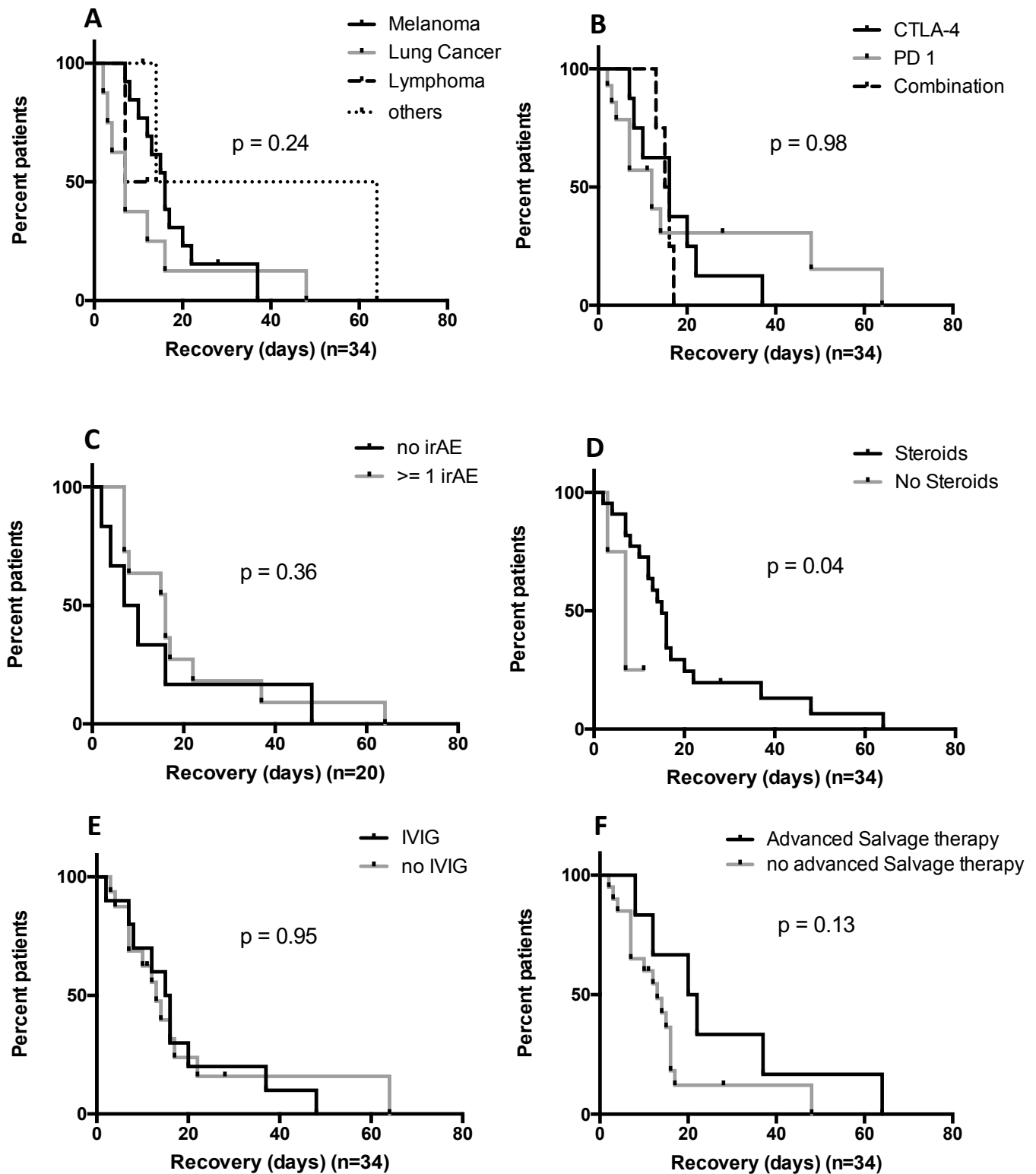

Supplement: Supplementary file 2 — Additional file 2. Kaplan-Meier curves for duration of neutropenia in days in a patient subpopulation Diseases (A) ICI Treatment (B) other immune-related adverse events = irAE (C). Neutropenia therapy with steroids (D), IVIG (+ Steroids) (E) and other secondary salvage therapy (+/− IVIG) (F). Significance threshold was defined as < 0.05. [file 12885_2020_6763_MOESM2_ESM.pdf]

# Additional file 3

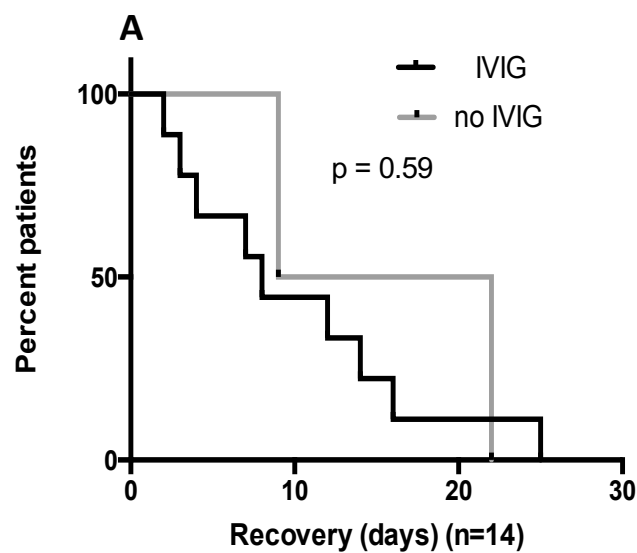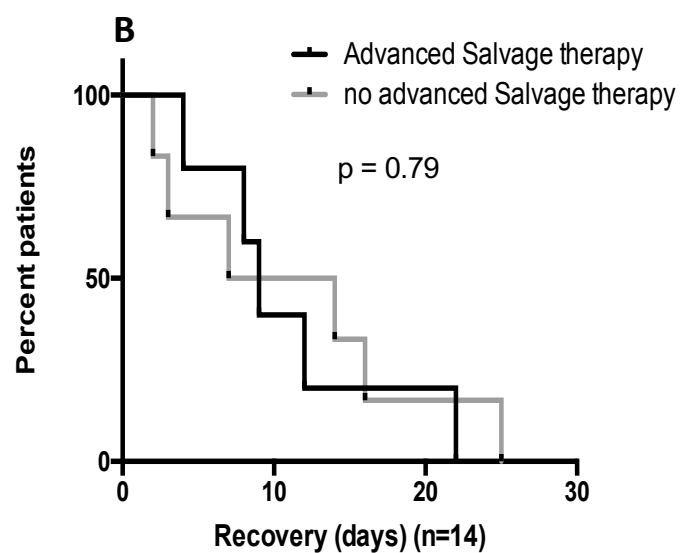

Supplement: Supplementary file 3 — Additional file 3 Kaplan-Meier curves for response of salvage treatments in days for IVIG alone (A) other salvage therapy (+/−) IVIG (B). A p-value of < 0.05 was considered significant. [file 12885_2020_6763_MOESM3_ESM.pdf]
